# A Recyclable Magnetic Aminated Lignin Supported Zr-La Dual-Metal Hydroxide for Rapid Separation and Highly Efficient Sequestration of Phosphate

**DOI:** 10.3390/molecules28072923

**Published:** 2023-03-24

**Authors:** Enmin Zong, Xuanren Wang, Lirong Zhang, Jiayao Yang, Xiaohuan Liu

**Affiliations:** 1 College of Life Science, Zhejiang Provincial Key Laboratory of Plant Evolutionary Ecology and Conservation, Taizhou University, 1139 Shifu Street, Taizhou 318000, China; 2 School of Earth Science and Engineering, Nanjing University, Nanjing 210093, China; 3 School of Biological and Chemical Engineering, Zhejiang University of Science and Technology, Hangzhou 310023, China

**Keywords:** lignin, magnetic adsorbent, Zr hydroxides, La hydroxides, phosphate, recycle

## Abstract

The application of lignin-based adsorbents in the efficient removal of phosphate from wastewater has attracted much attention and been intensively studied in recent years. However, most currently reported lignin-based adsorbents are difficult to recover and recycle. Herein, we have developed a recyclable, nanostructured bio-adsorbent, poly(ethyleneimine) (PEI)-modified lignin (LG) integrated with Fe_3_O_4_ and Zr-La dual-metal hydroxide (LG-NH_2_@Fe_3_O_4_@Zr-La), by the Mannich reaction followed by the chemical coprecipitation method. Multilayer adsorption existed on the surface of LG-NH_2_@Fe_3_O_4_@Zr-La based on the isotherm fitting curve, and its adsorption capacity reached 57.8 mg P g^−1^, exhibiting a higher phosphate uptake than most reported metallic oxide-based composites. The adsorption process was dominated by inner-sphere complexation of ligand-exchange and electrostatic interactions. Moreover, LG-NH_2_@Fe_3_O_4_@Zr-La exhibited excellent selectivity against coexisting anions, and the adsorption was more efficient under acidic conditions. When the phosphate concentration was 2.0 mg P L^−1^, the removal efficiency of phosphate reached 99.5% and the residual concentration was only 10 μg P L^−1^, which meets the United States Environmental Protection Agency (USEPA) standard for eutrophication prevention. In addition, the LG-NH_2_@Fe_3_O_4_@Zr-La displayed excellent reusability, maintaining 91.8% of removal efficiency after five cycles. Importantly, owing to the magnetic properties of the loaded Fe_3_O_4_, the resulting composite could be separated within 30 s under an external magnetic field. Thus, the separable and recyclable biobased magnetic adsorbent developed in this work exhibited promising application in phosphate capture from real sewage. This research study provides a new perspective for lignin valorization in lignocellulose biorefineries and establishes an approach for developing an economical and efficient bio-adsorbent for phosphate removal from wastewater.

## 1. Introduction

Water quality issues are a major challenge in promoting sustainable development worldwide [1,2]. Eutrophication is an increasingly global problem that can cause several adverse environmental impacts, such as anoxia and death of aquatic animals. Since phosphorus is one of the main causes of eutrophication [2], the concentration of phosphate for stringent discharge is less than 0.05 mg L^−1^ recommended by the United States Environmental Protection Agency (USEPA). Therefore, it is of great significance to explore an efficient strategy for phosphate removal from wastewater. Chemical precipitation, biological treatment, ion exchange and adsorption have been investigated for phosphate removal from aqueous solution by many researchers [3]. Among these, the adsorption process is considered to be the most promising choice due to its simple operation, convenient recyclability and high reliability. In recent years, biomass-derived adsorbents have attracted increasing attention in the field of pollution removal because of their sustainable resources, biocompatibility and low environmental impact [1,4]. In addition, abundant reactive groups on the surfaces of biomass enable functionalization, thereby enhancing the adsorption capacity towards target pollutants [5,6]. Therefore, developing effective bio-adsorbent from an abundant and sustainable biomass is of great importance for phosphate removal.

Biomass materials mainly include cellulose, hemicelluloses, chitosan, agricultural wastes and lignin. Among them, lignin is the second most abundant biomass component on Earth, and approximately 70 million tons are produced by paper mills worldwide every year [7,8,9,10]. Moreover, lignin has become a potential bio-adsorbent with excellent adsorption performance owing to its three-dimensional network structure and oxygen-containing functional groups [2,11,12]. Thus, developing phosphate bio-adsorbents using lignin has attracted increasing attention [13,14,15,16]. However, natural lignin often shows low efficiency in phosphate sequestration due to the lack of anion binding sites and water solubility [14]. The incorporation of metal components, such as Fe, Zr and La, into the lignin structure has been demonstrated to improve the adsorption capacity of phosphate. In the study of Luo et al. [15], an eco-friendly and low-cost lignin-based adsorbent was prepared through Fe(III) chelation to form a coordinate linkage on a lignin surface modified by triethylenetetramine based on the Mannich reaction and showed a high phosphate removal efficiency of 90%. Meanwhile, in our previous work, a lignin-based nanoadsorbent (AL-PEI-La) was developed by loading nano-La(OH)_3_ onto a lignin surface aminated by the grafting modification of poly(ethyleneimine) (PEI) in advance [17]. The AL-PEI-La nano-adsorbent showed high adsorption capacity and fast phosphate removal. Although significant progress has been made in the improvement of phosphate removal, these lignin-related adsorbents are commonly difficult to separate from solution, which limits their practical application [18].

To overcome the above problem, it was reported that magnetic media (e.g., Fe_3_O_4_) were added to the powder materials to form magnetic composites, which could easily be separated from aqueous solution by an external magnetic field. Compared with centrifugation and filtration, magnetic separation has several advantages, including high efficiency, low cost and green operation [18]. Therefore, magnetic media were introduced to the lignin adsorbents to generate magnetic composites [18,19]. For example, Tan et al. [20] reported a lignin-based magnetic bio-adsorbent through in-situ assemble of magnetic Fe_3_O_4_ on lignin and applied it to the capture of heavy metal ions from wastewater. The prepared magnetic lignin-based adsorbent was demonstrated to be easily recovered from aqueous solution. In addition, it was reported that bimetallic oxide nanocomposites exhibited a higher phosphate adsorption capacity than monometallic oxide due to the enhancement of ligand exchange, protonation and electrostatic interactions [21,22,23]. For instance, Du et al. [21] reported the maximum adsorption capacity of 61.31 mg P g^−1^ onto the bimetallic oxide adsorbent La-Zr-D201, which exceeded the adsorption capacity of the monometallic adsorbents DLa-201 and Zr-D201. Since lanthanum and zirconium oxides exhibited superior adsorption efficiency [24,25], it can be anticipated that phosphate-targeted adsorbents for effective phosphate sequestration may be developed using Zr-La binary metal (hydro) oxides. Inspired by the above intuition, the combined decoration of bimetallic oxides (Zr, and La) and magnetic media (Fe_3_O_4_) onto lignin is expected to greatly improve its phosphate adsorption performance and confer excellent recoverability under an external magnetic field. However, correlative studies on magnetically separable phosphate-targeted adsorbents have rarely been reported until now.

**Figure 1:**
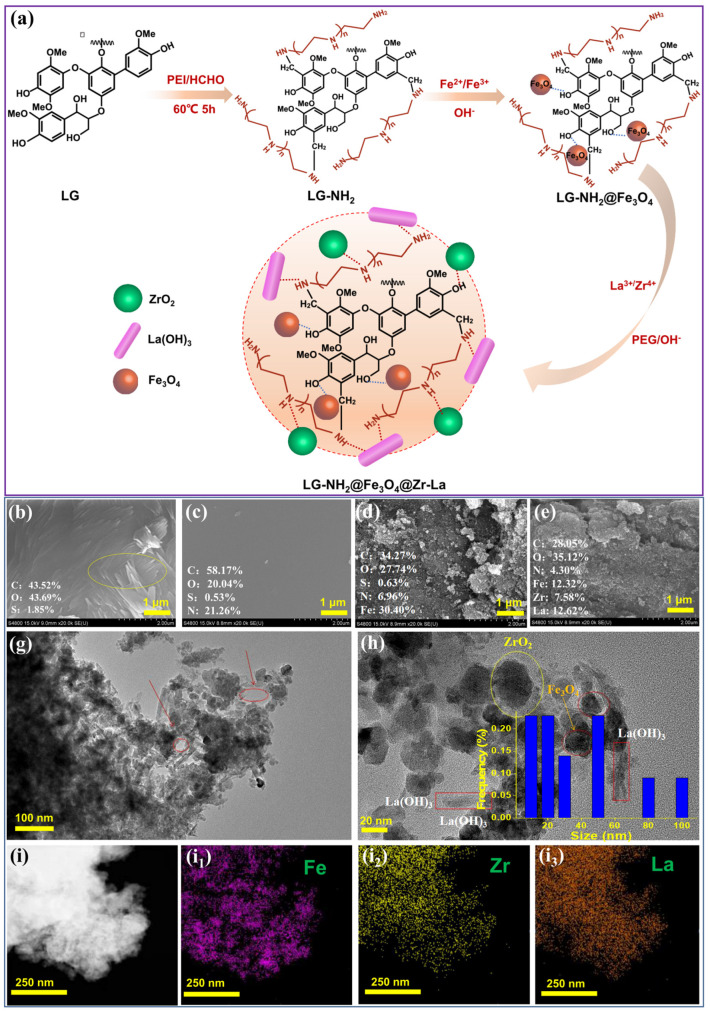
(**a**) Schematic drawing of experimental procedure adopted for the preparation, and SEM images for (**b**) LG, (**c**) LG-NH_2_, (**d**) LG-NH_2_@Fe_3_O_4_ and (**e**) LG-NH_2_@Fe_3_O_4_@Zr-La, and (**g**,**h**) TEM micrograph of LG-NH_2_@Fe_3_O_4_@Zr-La, showing the dispersion and phase sizes of particles and (**i**) TEM images for LG-NH_2_@Fe_3_O_4_@Zr-La and its elemental distribution maps of (**i_1_**) Fe, (**i_2_**) Zr and (**i_3_**) La.

Based on the above considerations, this study aims to prepare a novel magnetically recyclable lignin-based nano-adsorbent (LG-NH_2_@Fe_3_O_4_@Zr-La) via the Mannich reaction followed by the chemical coprecipitation method and apply it to phosphate removal. To improve the bonding between lignin and bimetallic oxides (Zr and La), polyethylenimine (PEI) with a number of amino functional groups was grafted onto lignin to form an aminated lignin via the Mannich reaction (Figure 1a), which can facilitate the loading of bimetallic oxides onto the aminated lignin. The morphological and chemical structures of LG-NH_2_@Fe_3_O_4_@Zr-La adsorbent were characterized by a variety of techniques. The phosphate adsorption properties of the adsorbents were investigated with varying initial phosphate concentrations, contact time, initial pH values, coexisting anions and ionic strengths. The reusability of the adsorbent was also evaluated, and the adsorption mechanism was illustrated. This study provides a novel sustainable strategy for the development of highly efficient, easy separable, recyclability bio-based adsorbents for removing phosphate, boosting the efficient utilization of industrial waste lignin.

## 2. Results and Discussion

### 2.1. Characterization of the LG-NH_2_@Fe_3_O_4_@Zr-La Adsorbent

#### 2.1.1. SEM and TEM Analysis

Figure 1b–e show SEM images of LG, LG-NH_2_, LG-NH_2_@Fe_3_O_4_ and LG-NH_2_@Fe_3_O_4_@Zr-La. Some wrinkles were observed on the surface of LG, as shown in the yellow circle of Figure 1b. After PEI grafting, the surface of LG-NH_2_ became relatively smooth, as shown in Figure 1c and Figure S1, indicating successful chemical cross-linking between EL and PEI. The as-prepared LG-NH_2_ was insoluble in water, mainly because abundant -NH-/-NH_2_ groups in PEI greatly increased intramolecular and intermolecular hydrogen bonding [25]. As shown in Figure 1d,e, a portion of Fe_3_O_4_ nanoparticles were dispersed on the surface of LG-NH_2_. Moreover, the corresponding EDX results indicated that the surface of LG mainly contained C, O and S elements, while 21.26 wt% of N element was detected after PEI grafting. The obvious decrease in C content was attributed to the incorporation of Fe (30.40 wt%) after the loading of Fe_3_O_4_. The LG-NH_2_@Fe_3_O_4_@Zr-La was composed of C (28.05 wt%), O (35.12 wt%), N (4.30 wt%), Fe (12.32 wt%), Zr (7.58 wt%) and La (12.62 wt%). Moreover, as shown in Figure S2, the color of lignin also changed during its functionalization. As shown in Figure 1g, a loose and porous structure could be clearly observed from the TEM image of LG-NH_2_@Fe_3_O_4_@Zr-La, which may provide its high adsorption capability for phosphate. Moreover, the LG-NH_2_@Fe_3_O_4_@Zr-La composite exhibited some rod-like or amorphous nanoparticles. The diameter of the magnetic particles ranged from ~10 nm to ~100 nm, with an average particle size of 39 nm (Figure 1h). As shown in Figure 1i_1_–i_3_, the elemental mapping of LG-NH_2_@Fe_3_O_4_@Zr-La showed good dispersion of Fe, Zr and La in the target product, indicating the successful immobilization of Fe, Zr and La species onto LG-NH_2_. The above results confirmed the successful preparation of the LG-NH_2_@Fe_3_O_4_@Zr-La adsorbent.

#### 2.1.2. XPS, XRD and FTIR Analysis

**Figure 2:**
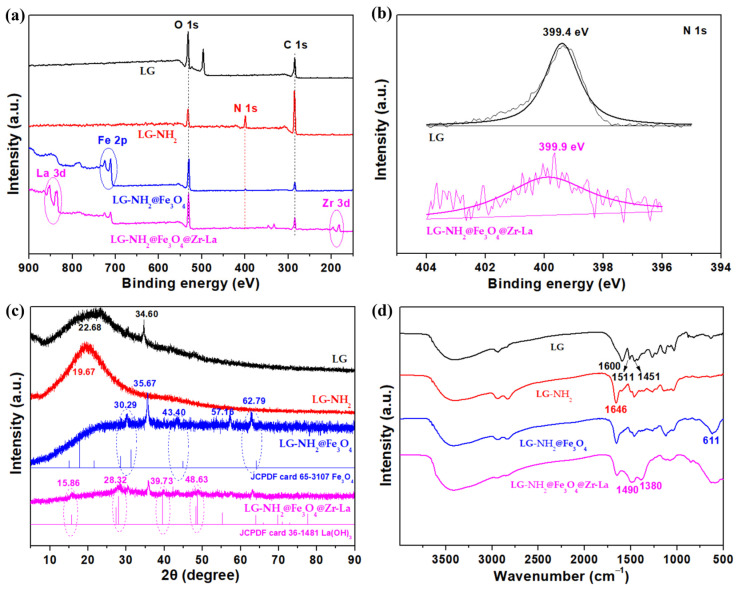
XPS spectra (**a**) of LG, LG-NH_2_, LG-NH_2_@Fe_3_O_4_ and LG-NH_2_@Fe_3_O_4_@Zr-La, and XPS_N 1S_ spectra (**b**) of LG and LG-NH_2_@Fe_3_O_4_@Zr-La, and XRD (**c**) and FTIR (**d**) spectra of LG, LG-NH_2_, LG-NH_2_@Fe_3_O_4_ and LG-NH_2_@Fe_3_O_4_@Zr-La.

The elemental composition and chemical bonds of LG-NH_2_@Fe_3_O_4_@Zr-La were determined by XPS analysis [26]. As shown in Figure 2a, the XPS survey of LG exhibited obvious signals of carbon and oxygen elements. After PEI functionalization, a new N 1s peak was observed in the spectrum of LG-NH_2_, indicating the successful grafting of PEI onto the LG. In addition, a Fe 2p peak appeared in the spectrum of LG-NH_2_@Fe_3_O_4_, implying the successful loading of Fe_3_O_4_ onto aminated lignin. The peaks of La 3d and Zr 3d were clearly observed in the spectrum of LG-NH_2_@Fe_3_O_4_@Zr-La. As shown in Figure 2b, the binding energy of N 1s shifted slightly from 399.4 eV to 399.9 eV, probably resulting from the coordination with Zr and La. Thus, the above results indicated that the lignin was successfully functionalized by PEI, Fe_3_O_4_ and Zr-La dual-metal hydroxide.

Furthermore, X-ray diffraction was employed to determine the crystalline structure of the as-prepared adsorbent. As shown in Figure 2c, the diffraction peak at 2θ of 22.68° was ascribed to the characteristic peak of LG. After PEI grafting, the peak position was shifted to a lower diffraction angle at 2θ of 19.67°, and the peak intensity increased a little. After Fe_3_O_4_ coating, the XRD pattern of LG-NH_2_@Fe_3_O_4_ could match with the standard cubic phase of Fe_3_O_4_ (JCPDF card no. 36-1481), including some distinctive peaks at 2θ of 30.29°, 43.40°, and 62.79°. The XRD pattern of LG-NH_2_@Fe_3_O_4_@Zr-La could match with the standard cubic phase of La(OH)_3_ (JCPDF card no. 36-1481), including four peaks at 2θ of 15.86°, 28.32°, 39.73°and 48.63°, corresponding to the (100), (101), (201), and (300) reflections of La(OH)_3_, respectively [27,28], but these characteristic peaks were weak, possibly due to the existence of some amorphous structures in the La(OH)_3_. Moreover, no characteristic peaks of ZrO_2_ crystals were observed, mainly due to the amorphous structure of ZrO_2_ impregnated in LG-NH_2_@Fe_3_O_4_ [21].

FTIR spectra were employed to characterize the chemical groups of LG-NH_2_@Fe_3_O_4_@Zr-La. As shown in Figure 2d, the stretching bands at 1600, 1511 and 1451 cm^−1^ were ascribed to aromatic rings of LG, and the peak at 1232 cm^−1^ was ascribed to the phenolic C-O groups. As for the LG-NH_2,_ it was observed that the absorption bands appeared at 3419 cm^−1^ (center position) and 1646 cm^−1^, which were attributed to symmetric and deformation vibration of -NH- group, respectively [29,30], indicating the successful grafting of PEI chains onto the LG structure. A wide peak at approximately 611 cm^−1^ was observed in the spectrum of LG-NH_2_@Fe_3_O_4_, which was attributed to the Fe-O bond vibration of Fe_3_O_4_ [31]. Obvious changes in the N-H vibrations were found in the spectrum of LG-NH_2_@Fe_3_O_4_@Zr-La due to the integration of Zr and La. Moreover, the absorption band at 1490 cm^−1^ was attributed to the asymmetric stretching mode of the CO_3_^2-^ group and caused by the existence of CO_2_ on the La(OH)_3_ surface [32]. In addition, LG-NH_2_@Fe_3_O_4_@Zr-La exhibited an obvious band at 1380 cm^−1^ due to O-H bending vibration, indicating the presence of hydroxyl groups (mostly Zr-OH).

#### 2.1.3. BET and VSM Analysis

**Figure 3:**
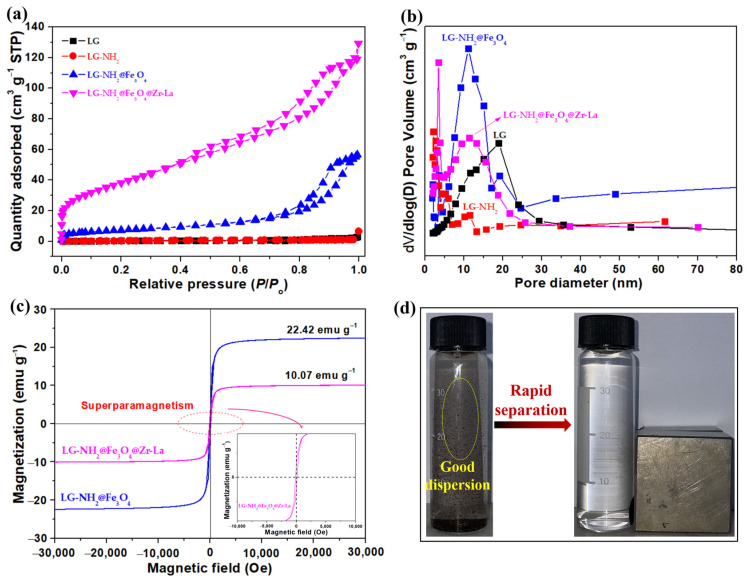
(**a**) N_2_ adsorption–desorption isotherms, (**b**) pore size distribution curves of LG, LG-NH_2_, LG-NH_2_@Fe_3_O_4_ and LG-NH_2_@Fe_3_O_4_@Zr-La, and (**c**) the hysteresis curves of LG-NH_2_@Fe_3_O_4_ and LG-NH_2_@Fe_3_O_4_@Zr-La, and (**d**) the photograph of rapid solid–liquid separation result of LG-NH_2_@Fe_3_O_4_@Zr-La.

The surface area and porous property of LG, LG-NH_2_, LG-NH_2_@Fe_3_O_4_ and LG-NH_2_@Fe_3_O_4_@Zr-La composites were characterized by N_2_ adsorption–desorption isotherms, as shown in Figure 3a. According to the IUPAC classification, all the samples were considered to be type IV isotherm, indicating the presence of an irregular mesoporous structure [33]. Compared with LG, LG-NH_2_ displayed slightly increased BET surface area (S_BET_) and total pore volume (V_Total_) after PEI functionalization. After the loading of Fe_3_O_4_, the S_BET_ of LG-NH_2_@Fe_3_O_4_ obviously increased to 26.46 m^2^ g^−1^, and the V_total_ increased from 0.0015 cm^3^ g^−1^ to 0.0868 cm^3^ g^−1^. The S_BET_ of LG-NH_2_@Fe_3_O_4_@Zr-La was further improved to 139.85 m^2^ g^−1^, which was attributed to the uniform loading of ZrO_2_ and La(OH)_3_. The average pore size of LG-NH_2_@Fe_3_O_4_@Zr-La (see Figure 3b) further indicated the presence of mesopores in the adsorbent, which would play a key role in the improvement of adsorption performance.

Figure 3c shows the magnetic hysteresis loops of LG-NH_2_@Fe_3_O_4_ and LG-NH_2_@Fe_3_O_4_@Zr-La. The saturation magnetizations of LG-NH_2_@Fe_3_O_4_ and LG-NH_2_@Fe_3_O_4_@Zr-La were 22.42 emu g^−1^ and 10.07 emu g^−1^, respectively. In addition, the coercivity and reminisce values of LG-NH_2_@Fe_3_O_4_@Zr-La were observed to be almost zero, indicating its superparamagnetic state [31]. It was observed that LG-NH_2_@Fe_3_O_4_@Zr-La particles exhibited a low saturation magnetization. The reason for the reduction in magnetization is on the one hand due to its low Fe_3_O_4_ content, but on the other hand, it may be attributed to the surface order/disorder interaction of the magnetic spin moment and the presence of a shell layer [34]. It could still be rapidly recovered from water under an external magnetic field within 30 s (Figure 3d). Therefore, LG-NH_2_@Fe_3_O_4_@Zr-La with a sensitive magnetic response could be easily separated from aqueous solution after the adsorption.

### 2.2. Phosphate Adsorption

#### 2.2.1. Phosphate Adsorption by Lignin-Based Materials

**Figure 4:**
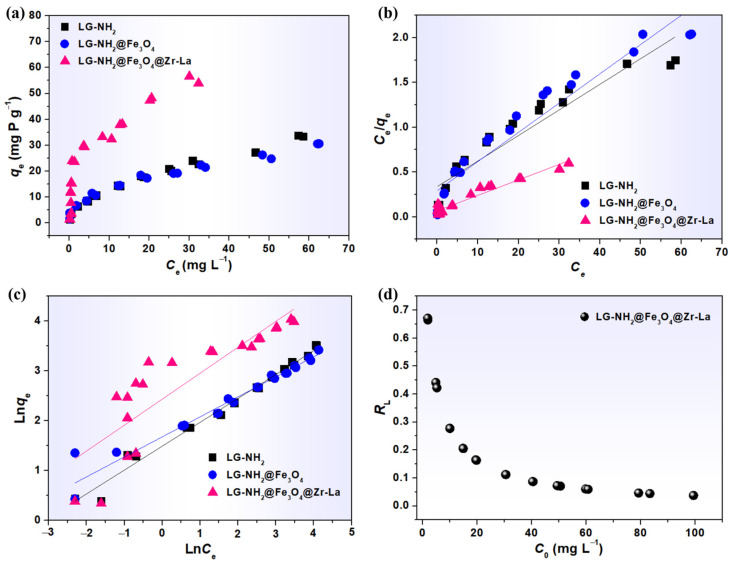
(**a**) Phosphate adsorption isotherms of LG-NH_2_, LG-NH_2_@Fe_3_O_4_ and LG-NH_2_@Fe_3_O_4_@Zr-La, and their fitting with (**b**) the Langmuir model and (**c**) the Freundlich model; (**d**) Langmuir separation factor (RL) plot for adsorption of phosphate on LG-NH_2_@Fe_3_O_4_@Zr-La.

The phosphate adsorption isotherms were examined at 25 °C to evaluate the performance of lignin-based adsorbents. As shown in Figure 4a, the pristine LG could be dissolved in an aqueous solution, which made it impossible to measure the phosphate adsorption capacity [14]. The LG-NH_2_ showed a certain adsorption capacity toward phosphate, mainly due to the interaction between positively charged quaternary ammonium groups and phosphate ions. After the loading of Fe_3_O_4_, the adsorption capacity of LG-NH_2_@Fe_3_O_4_ decreased a little, probably because the coordination between amino groups and irons reduced the amount of free amino groups. The impregnated Zr and La species exhibited preferable adsorption toward phosphate through ligand exchange or metal-ligand interactions [23].

The adsorption isotherms were further analyzed by the Langmuir and Freundlich isotherm models. The main fitting parameters, such as qm and b, were calculated based on the intercept and slope of the linear plots of C_e_/q_e_ versus C_e_ (Figure 4b), and the relative results are shown in Table 1.

It can be seen that the Freundlich model with a higher correlation coefficient (R^2^ = 0.9861) could better describe phosphate adsorption on the LG-NH_2_. In contrast, the Langmuir fitting result of LG-NH_2_@Fe_3_O_4_ (R^2^ = 0.9448) was consistent with the experimental data, indicating that the adsorption process could be considered to be monolayer adsorption. In addition, the maximum phosphate adsorption capacity of LG-NH_2_@Fe_3_O_4_@Zr-La reached 57.8 mg P g^−1^, much higher than those of LG-NH_2_ and LG-NH_2_@Fe_3_O_4_, according to the Langmuir isotherm model, which could be attributed to the combined effects of -NH_2_, ZrO_2_ and La(OH)_3_ [19]. Moreover, the separation factor constant (RL) is commonly applied to reflect the selectivity toward phosphate, and could be calculated from Equation (S1). As shown in Figure 4d, the RL values of LG-NH_2_@Fe_3_O_4_@Zr-La (0.0160–0.245) were between 0 and 1; accordingly, the value n in the Freundlich model (2.55) was between 1 and 10, indicating preferable adsorption toward phosphate ions.

Furthermore, bimetal materials for phosphate removal are summarized in Table 2; a performance comparison was conducted with the LG-NH_2_@Fe_3_O_4_@Zr-La composite [21,22,35,36,37,38,39]. It can be seen that the LG-NH_2_@Fe_3_O_4_@Zr-La composite exhibited a higher phosphate uptake than most metallic-oxide-based composites and showed a relatively good cycle regeneration efficiency, indicating its potential application as a new-generation sustainable adsorbent for phosphate removal from wastewater.

#### 2.2.2. Adsorption Kinetics

**Figure 5:**
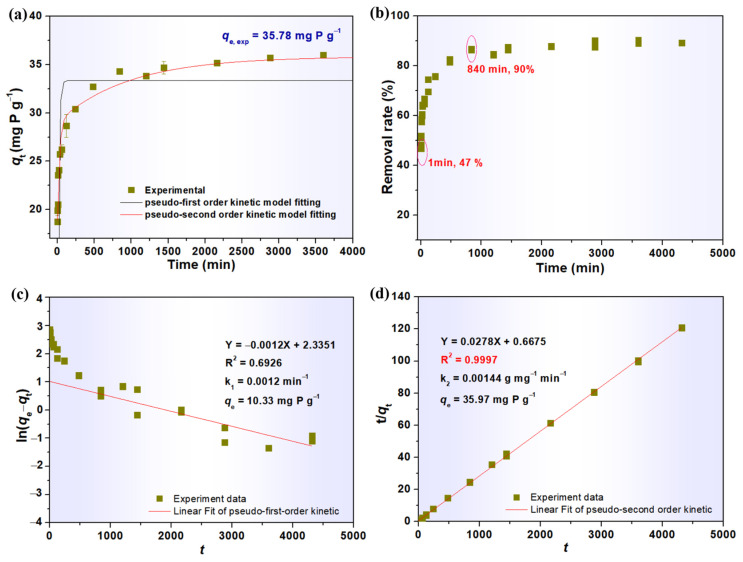
(**a**) Kinetic process and non-linear forms of pseudo-first-order and pseudo-second-order; (**b**) effect of contact time on phosphate removal rate; the linear plots results of pseudo-first-order model (**c**) and pseudo-first-order model (**d**).

The phosphate removal of LG-NH_2_@Fe_3_O_4_@Zr-La was investigated with different contact times to explore the adsorption kinetics. As shown in Figure 5a, the adsorption capacity of LG-NH_2_@Fe_3_O_4_@Zr-La gradually increased with the increase of the initial concentration, mainly due to an increase in the number of active sites for adsorption [17]. In addition, the LG-NH_2_@Fe_3_O_4_@Zr-La exhibited a rapid adsorption rate with 47% of phosphate removal during the first 1 min of adsorption. Moreover, the adsorption capacity reached 90% of the maximum adsorption capacity within 840 min, indicating that the porous structure of LG-NH_2_@Fe_3_O_4_@Zr-La could be conducive to the mass transfer and fast diffusion during the phosphate adsorption. The pseudo-first-order and pseudo-second-order kinetic models were used to fit the experiment data to better elucidate the adsorption mechanism of phosphate on the LG-NH_2_@Fe_3_O_4_@Zr-La composite.

As shown in Figure 5c,d, the phosphate adsorption of the LG-NH_2_@Fe_3_O_4_@Zr-La could be described by the pseudo-second-order model due to a larger correlation coefficient (R^2^ > 0.9997). In addition, the theoretical (*q*_e,cal_) values calculated from the pseudo-second-order model were more consistent with experimental values (*q*_e,exp_). These results indicated that the adsorption behavior of LG-NH_2_@Fe_3_O_4_@Zr-La followed the second-order kinetic model and was mainly governed by the chemisorption process [17].

#### 2.2.3. Effect of Temperature and Adsorption Thermodynamics

**Figure 6:**
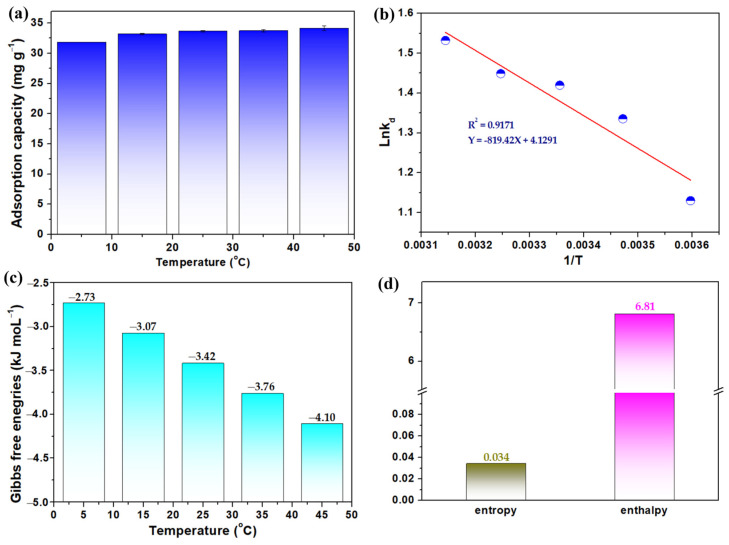
Effect of temperature on phosphate adsorption (**a**), and the plot of 1/T (k^−1^) versus lnk_d_ (**b**) and plot of ΔG0 versus T (**c**), and the calculated values of entropy and enthalpy (**d**).

Adsorption thermodynamics of LG-NH_2_@Fe_3_O_4_@Zr-La were investigated at different temperatures of 5, 15, 25, 35, and 45 °C. As shown in Figure 6a, the amount of phosphate adsorbed by LG-NH_2_@Fe_3_O_4_@Zr-La increased with the increase of temperature, mainly due to an increase in the frequency of collisions between the adsorbent and phosphate ions [40]. As can be seen from the plots of the distribution coefficient k_d_ versus T in Figure 6b, the k_d_ increased with the increase of temperature, indicating its endothermic process [40]. According to Equation (7), the values of ΔH° and ΔS° were calculated from the slope and intercept of the plot of ln*k*_d_ versus 1/T.

The overall changes in Gibbs free energy during the adsorption process were negative at 5, 15, 25, 35, and 45 °C, indicating an endothermic character (Figure 6c). The absolute value of ΔG° increased with increasing temperature, indicating that a high temperature was conducive to the adsorption process. The enthalpy change (ΔH° = 6.81 kJ mol^−1^) further indicated that the adsorption was endothermic in nature [17,40]. As shown in Figure 6d, the entropy change (ΔS° = 0.034 J K^−1^ mol^−1^) suggested that the randomness at the solid–solution interface increased during phosphate adsorption on the LG-NH_2_@Fe_3_O_4_@Zr-La.

#### 2.2.4. Effect of Coexisting Ions

**Figure 7:**
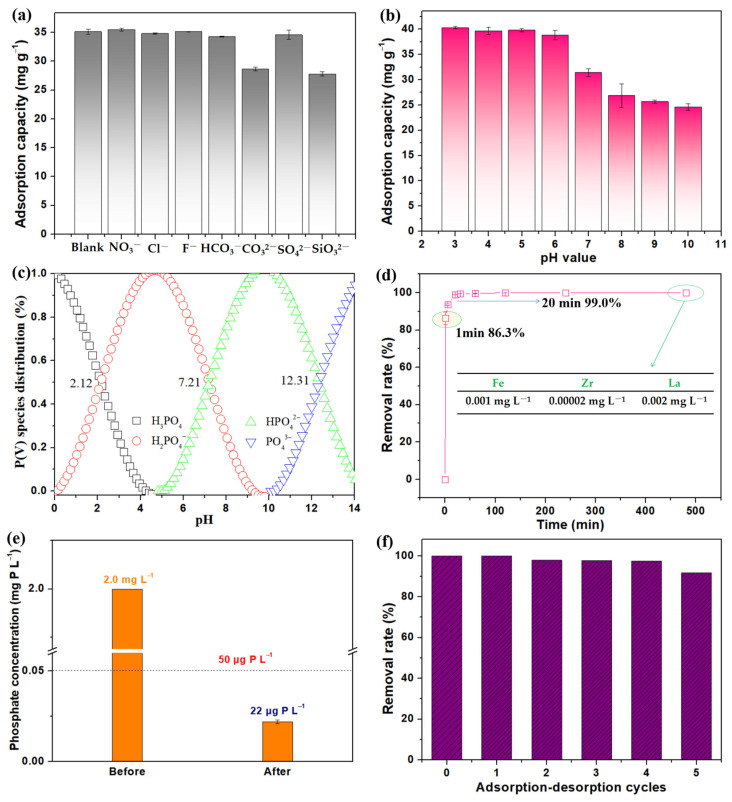
Effects of coexisting anion (**a**) and pH value (**b**) on phosphate adsorption, and (**c**) phosphate species distribution as a function of pH values, (**d**) effect of contact time on the phosphate removal rate in the low concentration phosphate, (**e**) changes of phosphate concentrations before and after adsorption, and (**f**) cycle adsorption and regeneration of batch experiments.

It is well known that industrial and domestic wastewater contains a variety of harmful anions, which would affect the actual adsorption of phosphate by adsorbents [25]. Thus, the selectivity of the LG-NH_2_@Fe_3_O_4_@Zr-La toward phosphate was investigated in the presence of the coexisting anions, including SiO_3_^2−^, CO_3_^2−^, SiO_3_^2−^, F^−^, Cl^−^, HCO_3_^−^, SO_4_^2−^, and NO_3_^−^. As shown in Figure 7a, the presence of most coexisting anions exerted no obvious influence on the adsorption capacity of phosphate. However, the phosphate adsorption capacity decreased considerably in the presence of SiO_3_^2−^, mainly because the addition of SiO_3_^2−^ with the largest hydrolysis constant showed the highest initial pH. The excellent selectivity of LG-NH_2_@Fe_3_O_4_@Zr-La for phosphate adsorption was mainly due to the formation of inner-sphere complexes with a strong affinity between LG-NH_2_@Fe_3_O_4_@Zr-La and phosphate in the aqueous solution.

#### 2.2.5. Effect of pH Value

Figure 7b presents the influence of pH on the phosphate uptake by LG-NH_2_@Fe_3_O_4_@Zr-La at an initial phosphate concentration of 50.0 mg P L^−1^. The phosphate adsorption amount of LG-NH_2_@Fe_3_O_4_@Zr-La reached 40.33 mg P g^−1^ at pH 3.0, and maintained a high level in the pH range of 3.0–6.0. When the pH changed to alkaline conditions (7.0–10.0), the phosphate uptake decreased to 24.65 mg P g^−1^ at pH 10.0. The zero point of charge (pH_ZPC_) of the LG-NH_2_@Fe_3_O_4_@Zr-La was detected as 3.56. When the pH of the solution was below pH_ZPC_, the adsorbent surface was positively charged, and vice versa. In addition, the species of phosphate was different with varying pH from 3.0 to 10.0, as expressed in the equations (Figure 7c). Monovalent dihydrogen phosphate was the dominant species in the aqueous solution with a pH of 2.13~7.20, indicating that LG-NH_2_@Fe_3_O_4_@Zr-La displayed a strong affinity to the single charged phosphate species (H_2_PO_4_^−^). When the pH of the solution increased from 7.20 to 10.0, the predominant form of phosphate species changed to hydrogen phosphate (HPO_4_^2−^). Therefore, as the pH increased, the negative charged surface of the adsorbent would repel phosphate ions (PO_4_^3−^, HPO_4_^2−^, and H_2_PO_4_^−^). Besides, the phosphate anion might compete with OH− under alkaline conditions, resulting in a low adsorption capacity of LG-NH_2_@Fe_3_O_4_@Zr-La in the pH range of 7.0–10.0 [23]. These results inspired the regeneration of LG-NH_2_@Fe_3_O_4_@Zr-La composite by the treatment of NaOH solution.

#### 2.2.6. Phosphate Removal from Low Concentration and Real Sewage

Since abnormal growths of microorganism may occur at very low phosphate concentrations, 50 μg P L^−1^ of phosphate was applied in some countries to prevent blooms of cyanobacteria. Thus, the phosphate removal property of the LG-NH_2_@Fe_3_O_4_@Zr-La adsorbent was carried out at an initial phosphate concentration of 2.0 mg P L^−1^. As shown in Figure 7d, the LG-NH_2_@Fe_3_O_4_@Zr-La exhibited a high adsorption rate and could remove 86.3% of phosphate in 1 min and 99.0% in 20 min. The residual phosphate concentration in the solution was 22 μg P L^−1^ after 20 min of adsorption by LG-NH_2_@Fe_3_O_4_@Zr-La, lower than the phosphate limit (50 μg P L^−1^). In addition, the leaching concentrations of Fe, Zr and La measured by ICP-MS were extremely low, indicating the structural stability of the LG-NH_2_@Fe_3_O_4_@Zr-La (Figure 7e).

In addition, the LG-NH_2_@Fe_3_O_4_@Zr-La was also applied to treat actual wastewater. As shown in Figure S3a,b, the phosphate concentration decreased from 1.50 or 1.0 to below 0.010 mg L^−1^ after the adsorption treatment by the LG-NH_2_@Fe_3_O_4_@Zr-La, which is lower than the level II water of environmental quality standards for Surface Water in China (GB3838-2002) [41].

#### 2.2.7. Regeneration of LG-NH_2_@Fe_3_O_4_@Zr-La

The service life of an effective adsorbent is crucial for commercial application [42]. To evaluate the reusability of the adsorbent, 1.0 g LG-NH_2_@Fe_3_O_4_@Zr-La was added into 800 mL phosphate solution with an initial concentration of 10 mg P L^−1^, and the adsorption was allowed for 24 h at 25 °C. After the adsorption, the LG-NH_2_@Fe_3_O_4_@Zr-La was desorbed with 2 M NaOH and washed with distilled water to recover for the next cycle. As shown in Figure 7d, the phosphate removal efficiency of the adsorbent was nearly 100% in the first regeneration, and 91.8% in the fifth regeneration, indicating an excellent recovery efficiency of the LG-NH_2_@Fe_3_O_4_@Zr-La.

### 2.3. Proposed Mechanisms for Phosphate Adsorption

The phosphate adsorption mechanism of the LG-NH_2_@Fe_3_O_4_@Zr-La could be elucidated on the basis of its specific structure [43]. As mentioned above, two types of active sites on the LG-NH_2_@Fe_3_O_4_@Zr-La were assumed, including hydroxyl groups on the loaded ZrO_2_ and La(OH)_3_ nanoparticles and positively charged protonated amino groups. Thus, phosphate adsorption was dominated by the specific adsorption of ZrO_2_ and La(OH)_3_ via ligand exchange by inner-sphere complexation and electrostatic attraction between phosphate anions and protonated amino groups [43]. The underlying adsorption mechanism for LG-NH_2_@Fe_3_O_4_@Zr-La was probed by the FTIR, XPS and Zeta potential measurements.

#### 2.3.1. FTIR Analysis

**Figure 8:**
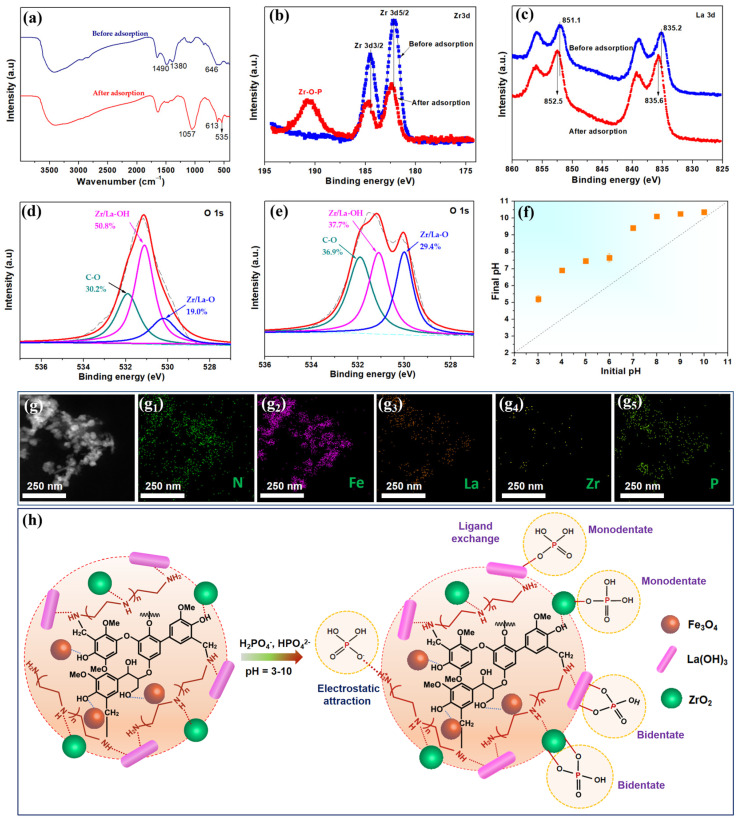
(**a**) FTIR spectra, (**b**) Zr 3d and (**c**) La 3d spectra of the LG-NH_2_@Fe_3_O_4_@Zr-La before and after phosphate adsorption, and (**d**,**e**) O 1s spectra before and after phosphate adsorption, and (**f**) the change of pH values in solution before and after adsorption. (**g**) TEM image for LG-NH_2_@Fe_3_O_4_@Zr-La after phosphate adsorption and its elemental distribution maps of (**g_1_**) N, (**g_2_**) Fe, (**g_3_**) La, (**g_4_**) Zr, and (**g_5_**) P, and (**h**) the possible mechanisms of phosphate adsorption by LG-NH_2_@Fe_3_O_4_@Zr-La.

Figure 8a shows the FTIR spectra of LG-NH_2_@Fe_3_O_4_@Zr-La before and after phosphate adsorption. A large number of amino groups were introduced into the target composite. Moreover, the phosphate adsorption capacity reached a maximum of 40.33 mg P g^−1^ at pH 3.0, mainly because the protonated amino groups produced a positive charged surface to promote phosphate adsorption [25]. Meanwhile, LG-NH_2_@Fe_3_O_4_@Zr-La exhibited an obvious band at 1380 cm^−1^ (O-H bending vibration) before the adsorption, indicating the presence of surface hydroxyl groups (mostly Zr-OH) [44]. In addition, the characteristic peaks at approximately 646 cm^−1^ were attributed to the bending O-H vibrations of La(OH)_3_ [17]. After phosphate adsorption, both peaks almost disappeared, and a new peak at 1057 cm^−l^ appeared due to symmetric and asymmetric stretching vibrations of the P-O bonds in HPO_4_^2−^ and H_2_PO_4_^−^ [45]. Furthermore, two new peaks at 535 cm^−1^ and 613 cm^−1^ were attributed to the bending vibrations of O-P-O [38]. Thus, the surface hydroxyl groups of ZrO_2_ and La(OH)_3_ in the adsorbent were probably replaced by the adsorbed phosphate ions. Besides, the pH of the equilibrium solution after the adsorption increased significantly compared with that of the initial solution (Figure 8f). The increase in the pH value was mainly due to the leaching out of OH− via ligand exchange. The above results demonstrated that the hydroxyl groups on the surface of LG-NH_2_@Fe_3_O_4_@Zr-La exerted a major role in the phosphate adsorption, which was replaced by phosphate in the adsorption process.

#### 2.3.2. XPS Analysis

Figure S4 shows the wide-scan XPS spectra of LG-NH_2_@Fe_3_O_4_@Zr-La before and after the adsorption. The characteristic peaks of La 3d (835.96 eV), Zr 3d (182.41 eV), Fe 2p (710.92 eV), O1s (531.23 eV), N 1s (399.56 eV), C 1s (284.80 eV) and P 2p (133.12 eV) were observed after phosphate adsorption, further suggesting that phosphate ions were adsorbed on LG-NH_2_@Fe_3_O_4_@Zr-La. Moreover, as shown in the high-resolution XPS spectra of P2p in Figure S5, the characteristic peak of P 2p (133.27 eV) shifted to a lower value compared with the basic spectrum of KH_2_PO_4_ (134.0 eV), indicating the formation of a strong chemical bond between phosphate and LG-NH_2_@Fe_3_O_4_@Zr-La. As shown in Figure 8b, the Zr 3d_5/2_ and 3d_3/2_ peaks of LG-NH_2_@Fe_3_O_4_@Zr-La exhibited binding energies of 182.8 eV and 185.2 eV, respectively [46]. These two peaks both shifted by 0.2 eV toward a lower binding energy after phosphate adsorption, mainly due to the formation of chemical bonds between Zr, La and phosphate by oxygen bridges. It is noteworthy that a peak at 191.57 eV was observed after phosphate adsorption, probably due to the formation of Zr-P complexes [47]. Meanwhile, it was observed, as shown in Figure 8c, that LG-NH_2_@Fe_3_O_4_@Zr-La exhibited higher binding energies after phosphate adsorption. In addition, as shown in Figure 8d,e, the O 1s spectra could be resolved into three overlapping peaks, including Zr/La-O at 530.1 eV, Zr/La-OH at 531 eV, and C-O at 532 eV, derived from the LG. Besides, the -OH percentage increased from 19.0% to 29.4%, which was attributed to the formation of O-P bonds on LG-NH_2_@Fe_3_O_4_@Zr-La. This finding confirmed that Zr/La-O functions participated in the phosphate adsorption process. Some large particles were observed in the SEM image of LG-NH_2_@Fe_3_O_4_@Zr-La after the adsorption, as shown in Figure S6, and the elemental composition (Figure S7) further demonstrated that the inner-sphere complexation was formed via ligand exchange reactions with the replacement of surface hydroxyl groups by phosphate ions [43].

#### 2.3.3. Zeta Potential Analysis

As shown in Figure S8, LG-NH_2_@Fe_3_O_4_@Zr-La displayed a pH_pzc_ value of 3.56. Therefore, phosphate adsorption of LG-NH_2_@Fe_3_O_4_@Zr-La was pH-dependent. When the pH of the solution was lower than 3.56, both ligand exchange and electrostatic attraction existed in the phosphate adsorption. However, when the pH of the solution was above 3.56, the phosphate adsorption was dominated by ligand exchange [48].

Based on the above results, Figure 8h illustrates the possible mechanisms of phosphate adsorption. Phosphates would be adsorbed on the surface of the adsorbent based on electrostatic interactions, ligand exchange and phosphate-magnetite complex formation. The diversity of adsorption mechanisms enabled LG-NH_2_@Fe_3_O_4_@Zr-La to be an efficient adsorbent for phosphate removal from aqueous solution.

## 3. Materials and Methods

### 3.1. Materials and Chemicals

The lignin (LG) used in the present work was purchased from Sigma-Aldrich Co. (St. Louis, MI, USA). Lanthanum nitrate hexahydrate (La(NO_3_)·6H_2_O, 99%), Zirconyl chloride octahydrate (ZrOCl_2_·8H_2_O, 99%), poly(ethyleneimine) (PEI, Mw 70000), ammonium iron (II)-sulfate hexahydrate (Fe(NH_4_)_2_(SO_4_)_2_·6H_2_O, 99.5%), ammonium ferric sulfate dodecahydrate (NH_4_Fe (SO_4_)_2_·12H_2_O, 99%), potassium dihydrogen phosphate (KH_2_PO_4_, 99.5%), potassium fluoride (KF, 99%), potassium nitrate (KNO_3_, 99%), potassium sulfate (K_2_SO_4_, 99%), potassium hydrogen carbonate (KHCO_3_, 99.5%), potassium carbonate (K_2_CO_3_, 99%), potassium chloride (KCl, 99.5%) were purchased from Aladdin Chemistry Co., Ltd. (Shanghai, China). Hydrochloric acid (HCl, 36%), sodium hydroxide (NaOH, 97%) and formaldehyde (HCHO, 37%) were purchased from Sinopharm Chemical Reagent Co., Ltd. (Shanghai, China).

### 3.2. Preparation of LG-NH_2_@Fe_3_O_4_@Zr-La Adsorbent

#### 3.2.1. Synthesis of LG-NH_2_

The LG-NH_2_ was synthesized by the Mannish reaction according to our previous work [17]. In brief, 5.0 g lignin (LG), 4.0 g formaldehyde, and 4.0 g polyethylenimine (PEI) were added to 100 mL distilled water. The mixture was mechanically stirred at a speed of 200 rp for 30 min, and the pH of the solution was adjusted to 10.0 using NaOH. The reaction was continued for 5 h at 60 °C. After that, the pH of the solution was adjusted to 3.0 with 1.0 M HCl to obtain precipitates. The precipitates were collected, rinsed with deionized water, and vacuum-dried for 24 h at 60 °C. Finally, the obtained product was denoted as LG-NH_2_.

#### 3.2.2. Preparation of LG-NH_2_@Fe_3_O_4_

The LG-NH_2_@Fe_3_O_4_ was synthesized as follows [49]. First, 6.8 g Fe(NH_4_)_2_(SO_4_)_2_·6H_2_O, and 10.4 g FeNH_4_(SO_4_)_2_·12H_2_O were dissolved into 500 mL distilled water, followed by the addition of 4.0 g LG-NH_2_ and ultrasonic dispersion for 0.5 h using an ultrasound bath at 40 kHz of ultrasound frequency and 150 W power. Subsequently, a certain amount of NaOH solution (2.0 M) was slowly dripped into the above solution to adjust the pH as 10.0. The mixed solution was stirred at 50 °C for 1 h, and the obtained precipitate was washed with deionized water three times. Black LG-NH_2_@Fe_3_O_4_ was obtained after drying under vacuum at 60 °C for 24 h.

#### 3.2.3. Preparation of LG-NH_2_@Fe_3_O_4_@Zr-La

The target product, LG-NH_2_@Fe_3_O_4_@Zr-La, was prepared via chemical precipitation method, as shown in Figure 1a. Specifically, 2.1652 g La(NO_3_)_3_ and 1.6112 g ZrOCl_2_ were dissolved into 200 mL distilled water, and then 2 g LG-NH_2_@Fe_3_O_4_ was added into the above mixture. The pH of the solution was adjusted to 10.0 with 1.0 M NaOH. After stirring at 60 °C for 2 h, the solution was allowed to continuously react at 25 °C for 24 h. The obtained grayish precipitate was separated, washed, and dried under vacuum at 60 °C for 12 h.

### 3.3. Phosphate Adsorption Evaluation of the LG-NH_2_@Fe_3_O_4_@Zr-La Adsorbent

Phosphate aqueous solution prepared with anhydrous KH_2_PO_4_ and deionized water was used for adsorption experiments. The adsorption isotherm of LG-NH_2_@Fe_3_O_4_@Zr-La was carried out varying the initial phosphate concentration from 2.0 to 100 mg P L^−1^ at 298 K. The adsorption kinetics of LG-NH_2_@Fe_3_O_4_@Zr-La was performed with an initial concentration of 50 mg P L^−1^ and an interval of 1.0~4320 min. The phosphate adsorption of LG-NH_2_@Fe_3_O_4_@Zr-La under different initial pH (3.0 to 10.0) was investigated in Teflon-lined screw-capped test tubes containing 50 mg LG-NH_2_@Fe_3_O_4_@Zr-La and 40 mL of 50 mg P L^−1^ phosphate solution at 298 K. The effect of temperature on the phosphate adsorption of LG-NH_2_@Fe_3_O_4_@Zr-La was also investigated at 5, 15, 25, 35, and 45 °C. Seven coexisting anions (Cl^−^, F^−^, SiO_3_^2−^, CO_3_^2−^, NO_3_^−^, SO_4_^2−^, HCO_3_^−^) at concentrations of 300 mg L^−1^ were applied to investigate the effect of competitive ions on phosphate adsorption at 25 °C. After adsorption, the suspension was filtered through a 0.45 μm filter and the concentration of phosphate was determined by UV-Vis spectrometer (PUXI, China). All the adsorption experiments were conducted twice to obtain the average values. The adsorption capacity (qe, mg P g^−1^) and removal rate (R, %) were calculated using Equations (1) and (2).

(1) $${\;q}_{e}=\frac{\left(C_{0}-C_{e}\right)V}{m}\;$$

(2) $$R=\frac{\left(C_{0}-C_{e}\right)}{C_{0}}\times 100\%$$

where *C*_0_ (mg P L^−1^), *C_e_* (mg P L^−1^), *V* (mL), and *m* (mg) are the initial concentration of phosphate in solution, equilibrium concentration of phosphate in solution, adsorbent mass and solution volume, respectively.

Adsorption kinetics at low concentration was conducted by immersing 50 mg LG-NH_2_@Fe_3_O_4_@Zr-La into 40 mL of 2.0 ppm phosphate aqueous solution. During adsorption, the solution was sampled at 1, 3, 5, 10, 20, 30, 60, 120, 240 and 480 min. After filtration through a 0.45 μm membrane syringe filter, the phosphate concentration in the sampled solution was determined by ICP-MS (Thermo Scientific, Waltham, MA, USA). After adsorption for 480 min, the leakage concentrations of Fe, Zr and La ions in the solution were determined by ICP-MS to evaluate the stability of LG-NH_2_@Fe_3_O_4_@Zr-La. The practical application of LG-NH_2_@Fe_3_O_4_@Zr-La in advanced water treatment was also conducted at 25 °C by adding 50 mg LG-NH_2_@Fe_3_O_4_@Zr-La to 40 mL of sewage water taken from Taizhou Huangyan North Control Sewage Treatment Plant. The reusability of LG-NH_2_@Fe_3_O_4_@Zr-La was investigated by adding 1.0 g sample to 800 mL of 10 mg P L^−1^ phosphate solution for 24 h at 25 °C. The adsorption–desorption process was performed for five cycles, and NaOH (2 M) solution was used as the regenerant. After each cycle, LG-NH_2_@Fe_3_O_4_@Zr-La was rinsed with deionized water to neutral pH to ensure similar adsorption conditions. The concentration of phosphate in the final solution was analyzed by UV-vis spectrophotometry (T6, Beijing Puxi Instrument Co., Beijing, China).

### 3.4. Data Analysis and Modeling

The adsorption isotherms were analyzed by the Langmuir (Equation (3)) and Freundlich isotherm (Equation (4)) models. The main fitting parameters, such as qm and b, were calculated based on the intercept and slope of the linear plots of *C*_e_/*q*_e_ versus *C*_e_ (Figure 4b), and the relative results were shown in Table 1.

(3) $$\mathrm{Langmuir}\;\mathrm{model}:\;\frac{C_{e}}{q_{e}}=\frac{1}{q_{m}b}+\frac{C_{e}}{q_{m}}$$

(4) $$\mathrm{Freundlich}\;\mathrm{model}:\;\ln qe=\mathrm{lnKf}+\frac{1}{n}lnCe$$

where *q*_e_ (mg g^−1^) is the amount of adsorbed phosphate per unit weight of adsorbent at equilibrium, *C*_e_ (mg L^−1^) is the equilibrium concentration of phosphate, b (L mg^−1^) is the Langmuir model constant, *q*_m_ (mg g^−1^) is the maximum adsorption capacity, KF is the Freundlich model constant, and 1/n is the heterogeneity factor.

As for the adsorption kinetics, the experimental data were fitted in the pseudo-first-order and pseudo-second-order models, which are described as the following equations.

(5) $$\log\left(q_{e}-q_{t}\right)=\log q_{e}-\frac{k_{1}t}{2.303}$$

(6) $$\frac{t}{qt}=\frac{1}{k_{2}qe^{2}}+\frac{t}{qe}$$

where *q*_e_ and *q*_t_ (mg g^−1^) represent the adsorbed amount (mg g^−1^) at equilibrium and time *t*, respectively. The rate constant *k*_1_ (g mg^−1^ min^−1^) in the pseudo-first-order model could be calculated from the slope of the linear log (*q*_e_—*q*_t_) as a function of *t* at different phosphate concentrations. The rate constant *k*_2_ (g mg^−1^ min^−1^) of the pseudo-second-order model could be calculated from the plots of (*t*/*q*_t_) against *t*.

The thermodynamic parameters, viz., standard Gibbs free energy change (ΔG°), standard enthalpy change (ΔH°), and standard entropy change (ΔS°), were determined according to Equations ((7)–(9)).

(7) $$\Delta G^{0}=-\mathrm{RTln}k_{d}$$

(8) $$\Delta G^{0}=\Delta H^{0}-T\Delta S^{0}$$

(9) $$\ln k_{d}=\frac{\Delta S^{0}}{R}-\frac{\Delta H^{0}}{RT}$$

where *k*_d_ represents the adsorption equilibrium constant (*q*_e_/*C*_e_). R is the gas constant of 8.314 J mol^−1^ K^−1^. T is the reaction temperature (K). ΔH° and ΔS°values can be calculated from the slope and intercept of the plot of ln*k*_d_ against T, respectively.

### 3.5. Structural Characterization of the LG-NH_2_@Fe_3_O_4_@Zr-La Adsorbent

The surface morphologies and chemical compositions of LG, LG-NH_2_, LG-NH_2_@Fe_3_O_4_ and LG-NH_2_@Fe_3_O_4_@Zr-La were detected on a Hitachi S-4800 field emission scanning electron microscope (SEM) coupled with an energy-dispersive X-ray spectrometry (EDX) detector at an accelerated voltage of 15,000 V. In addition, LG-NH_2_@Fe_3_O_4_@Zr-La was also observed with transmission electron microscopy (TEM, JEOL JEM-1230). X-ray photoelectron spectroscopy (XPS) spectra of LG, LG-NH_2_, LG-NH_2_@Fe_3_O_4_ and LG-NH_2_@Fe_3_O_4_@Zr-La were recorded on a Thermo ESCALAB 250 spectrometer. X-ray diffraction (XRD) patterns were collected in a 2θ range of 5–90 degrees at a scanning speed of 2 min^−1^ on a Bruker D8 Advance diffractometer using filtered Cu-Kα radiation. Fourier transform infrared (FTIR) spectra of LG, LG-NH_2_, LG-NH_2_@Fe_3_O_4_ and LG-NH_2_@Fe_3_O_4_@Zr-La were conducted on a Bruker Vector 22 FTIR spectrometer using the KBr pellet-pressing method. Nitrogen adsorption–desorption isotherms of LG, LG-NH_2_, LG-NH_2_@Fe_3_O_4_ and LG-NH_2_@Fe_3_O_4_@Zr-La were performed at 77 K on a Micromeritics ASAP 2460 M analyzer (Quantachrome, Boynton Beach, Florida, USA). Prior to the test, each sample was dried at 105 °C for 24 h. The BET specific surface area (S_BET_) and average pore size (dp) were analyzed based on Barrett-Joyner-Halenda (BJH) method. The hysteresis loops of LG-NH_2_@Fe_3_O_4_ and LG-NH_2_@Fe_3_O_4_@Zr-La were carried out on a physical property measurement system (PPMS-9, Quantum Design Inc., San Diego, CA, USA) under magnet fields in the range of −3~3 T.

## 4. Conclusions

In this work, a novel magnetic bio-adsorbent LG-NH_2_@Fe_3_O_4_@Zr-La for selective phosphate adsorption was prepared by the Mannich reaction, followed by chemical coprecipitation. The LG-NH_2_@Fe_3_O_4_@Zr-La adsorbent exhibited a large surface area of 139.85 m^2^ g^−1^, a total pore volume of 0.175 cm^3^ g^−1^, and a high phosphate adsorption capacity of 57.8 mg P g^−1^ according to the Langmuir mode. The phosphate adsorption kinetics confirmed the pseudo-second-order model, and the adsorption process was considered to be chemisorption through monolayer adsorption. The phosphate adsorption capacity was greatly affected by the pH of the solution and decreased with increasing pH. The presence of most coexisting ions exerted little influence on phosphate adsorption capacity, except for SiO_3_^2−^ and CO_3_^2−^. In addition, the LG-NH_2_@Fe_3_O_4_@Zr-La displayed excellent regeneration capability during five adsorption–desorption cycles. The Zr/La species were present in the forms of ZrO_2_ and La(OH)_3_ on the LG-NH_2_@Fe_3_O_4_, and the phosphate adsorption was possibly accomplished by the substitution of hydroxyl groups by phosphate species. Therefore, the LG-NH_2_@Fe_3_O_4_@Zr-La adsorbent with efficient adsorption performance is anticipated to be applied in the remediation of phosphate-contaminated water and also shows great potential as a novel bio-adsorbent for water treatment.

## Figures and Tables

**Table 1:** Equilibrium parameters for the Langmuir and Freundlich models.

Samples	Langmuir Model			Freundlich Model		
	b (L mg^−1^)	*q*_m_ (mg g^−1^)	R^2^	*K*_f_ (L mg^−1^)	n	R^2^
LG-NH_2_	0.085	34.97	0.9107	4.444	2.088	0.9861
LG-NH_2_@Fe_3_O_4_	0.112	30.58	0.9328	5.356	2.491	0.9589
LG-NH_2_@Fe_3_O_4_@Zr-La	0.259	57.80	0.9448	11.409	1.923	0.7577

**Table 2:** Comparison of phosphate adsorption performance on LG-NH_2_@Fe_3_O_4_@Zr-La with some bimetal adsorbents.

Absorbents	Temperature	pH	Recovery Efficiency	Adsorption Capacity (mg P g^−1^)	References
La-Zr-D201	T = 298 K	pH = 6.5 ± 0.3	Over 95% of original adsorption capacity (5 cycles)	61.31	Du et al. [21]
La-Zr@Fe_3_O_4_	T = 298 K	pH = 3	Over 90% removal efficiency (5 cycles)	49.3	Lin et al. [22]
SP-Zr-La	T = 298 K	pH = 3	Over 90% of original adsorption capacity (10 cycles)	45.2	Du et al. [23]
AMOCZ	T = 298 K	pH = 7	70.6% of original adsorption capacity (5 cycles)	7.56	Liu et al. [35]
Magnetic Fe-Zr binary oxide	T = 298 K,	pH = 4	66.7% of original adsorption capacity (5 cycles)	13.65	Long et al. [36]
Fe-Ti bimetal oxide	T = 293.15 K	pH = 6.8	82% of original adsorption capacity (5 cycles)	35.4	Lu et al. [37]
ACF-LaFe	T = 298 K	/	/	29.44	Liu et al. [38]
Fe_3_O_4_@La-Ce	T = 313 K	pH = 3	/	53.2	Han et al. [39]
LG-NH_2_@Fe_3_O_4_@Zr-La	T = 298 K,	pH = 6.0 ± 0.2	91.8% removal efficiency (5 cycles)	57.8	This work

## Data Availability

All the data have been included in the study.

## Supplementary Materials

The following supporting information can be downloaded at: https://www.mdpi.com/article/10.3390/molecules28072923/s1, Equation (S1): Calculation of *R*_L_; Figure S1: SEM images for (a) LG, (b) LG-NH_2_, (c) LG-NH_2_@Fe_3_O_4_, and (d) LG-NH_2_@Fe_3_O_4_@Zr-La; Figure S2: Digital images of different materials; Figure S3: Changes in phosphate concentrations of two sewage samples before and after treatment by LG-NH_2_@Fe_3_O_4_@Zr-La (ND: Not detected); Figure S4: The wide-scan XPS spectra of LG-NH_2_@Fe_3_O_4_@Zr-La before and after phosphate adsorption; Figure S5: XPS analysis of the P 2p spectral title; Figure S6: SEM image of the phosphate adsorbed-material; Figure S7: EDX spectrum of phosphate adsorbed-material; Figure S8: Zeta potential in response to pH change.
