# Supplementary material for: A Recyclable Magnetic Aminated Lignin Supported Zr-La Dual-Metal Hydroxide for Rapid Separation and Highly Efficient Sequestration of Phosphate
Source: Molecules. 2023 Mar 24;28(7):2923. doi: 10.3390/molecules28072923 (PMC10095728; doi:10.3390/molecules28072923)
Supplement: Supplementary file 1 [file molecules-28-02923-s001.zip › molecules-2279771-supplementary.pdf]

Supplementary Material

A Recyclable Magnetic Aminated Lignin Supported Zr-La Dual-Metal Hydroxide for Rapid Separation and Highly Efficient Sequestration of Phosphate

Enmin Zong [1,2], Xuanren Wang [1], Lirong Zhang [1], Jiayao Yang [3] and Xiaohuan Liu [1,*]

[1] College of Life Science, Zhejiang Provincial Key Laboratory of Plant Evolutionary Ecology and Conservation, Taizhou University, 1139 Shifu Street, Taizhou 318000, China; zongenmin@tzc.edu.cn (E.Z.); 2131340013@tzc.edu.cn (X.W.); 2131340030@tzc.edu.cn (L.Z.)
[2] School of Earth Science and Engineering, Nanjing University, Nanjing 210093, China
[3] School of Biological and Chemical Engineering, Zhejiang University of Science and Technology, Hangzhou 310023, China; jiayao_yang@zust.edu.cn
* Correspondence: liuxh@tzc.edu.cn

Equation:

$$R_L = \frac{1}{1 + bC_0} \quad (1)$$

where C_0 represents the initial concentration of phosphate in aqueous medium and b represents the Langmuir equilibrium constant. The value of R_L shows an indication for the feasibility of the adsorption process to proceed: irreversible ($R_L = 0$), suitable ($0 < R_L < 1$), linear ($R_L = 1$), or unsuitable ($R_L > 1$), respectively.

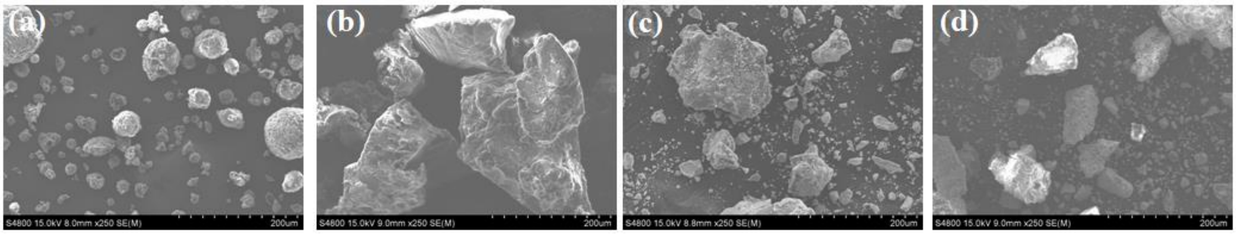

Figure S1. SEM images for (**a**) LG, (**b**) LG-NH_2, (**c**) LG-NH_2@Fe_3O_4, and (**d**) LG-NH_2@Fe_3O_4@Zr-La.

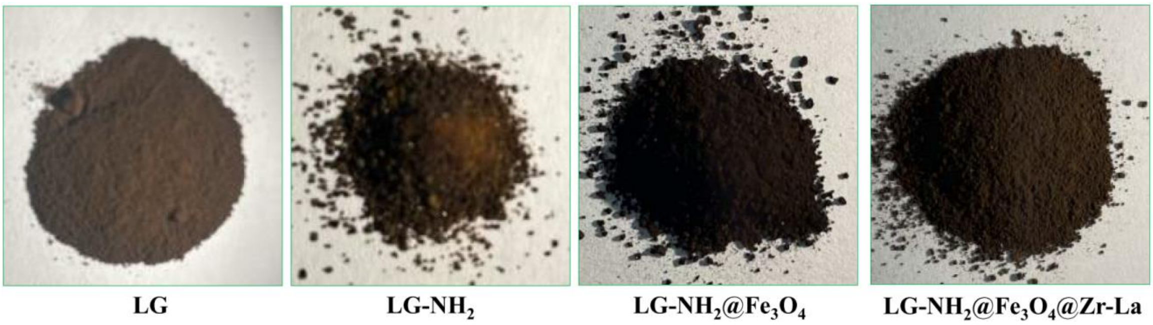

Figure S2. Digital images of different materials.

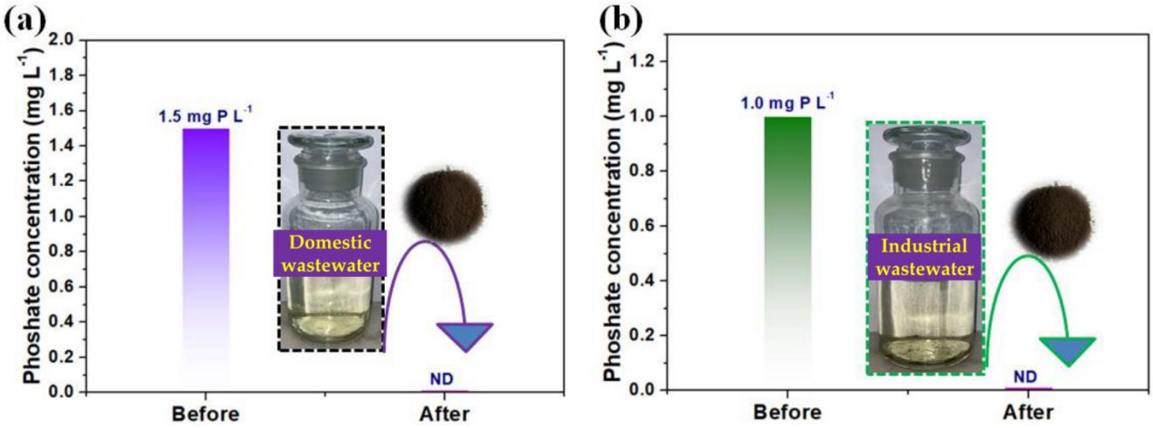

Figure S3. Changes in phosphate concentrations of two sewage samples before and after treatment by LG-NH_2@Fe_3O_4@Zr-La (ND: Not detected).

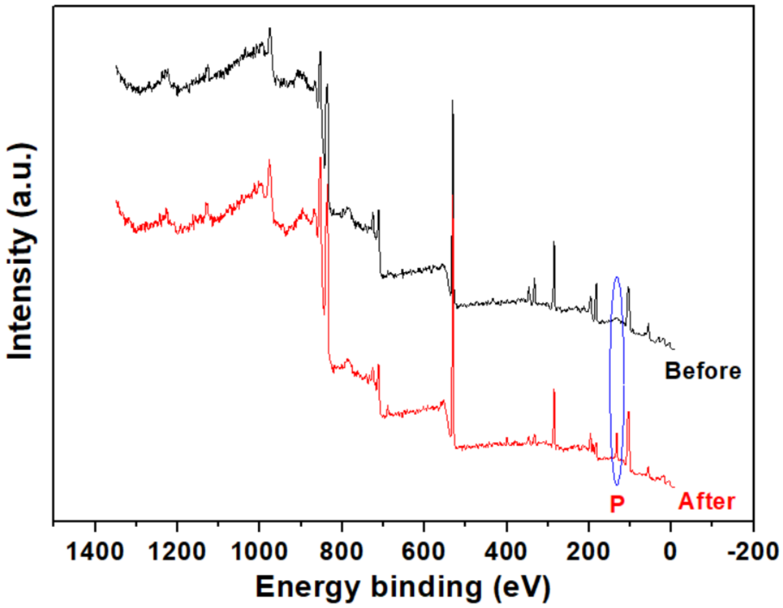

Figure S4. The wide-scan XPS spectra of LG-NH_2@Fe_3O_4@Zr-La before and after phosphate adsorption.

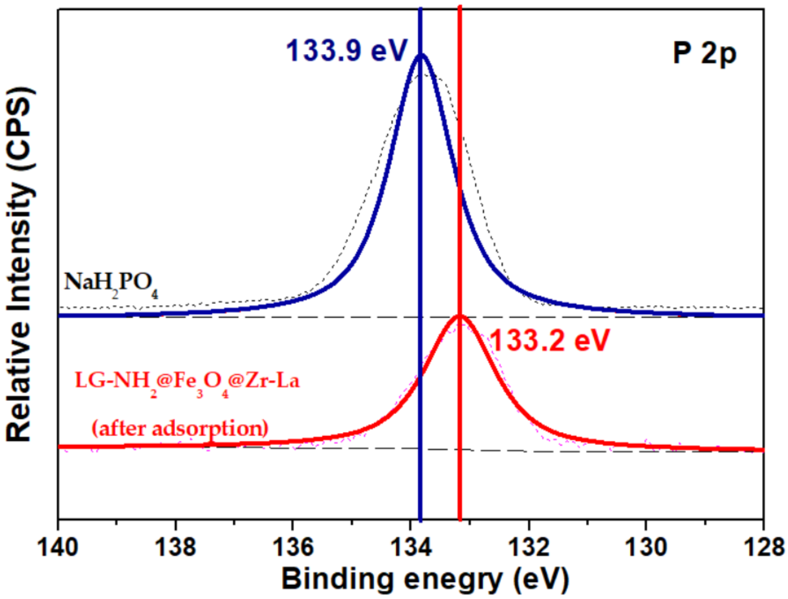

Figure S5. XPS analysis of the P 2p spectral.

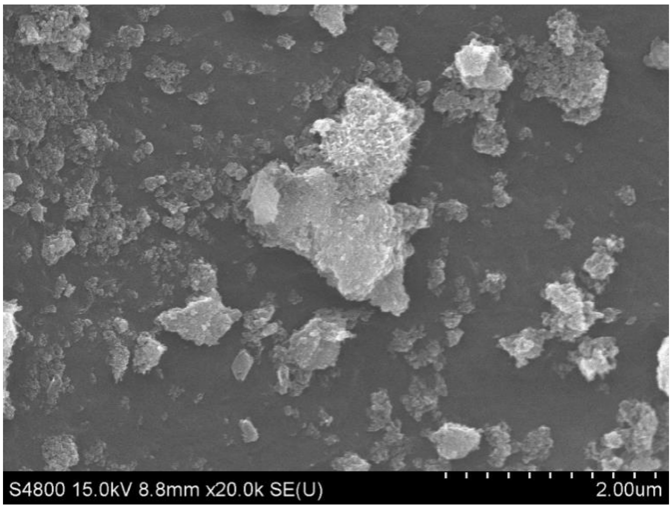

Figure S6. SEM image of the phosphate adsorbed-material.

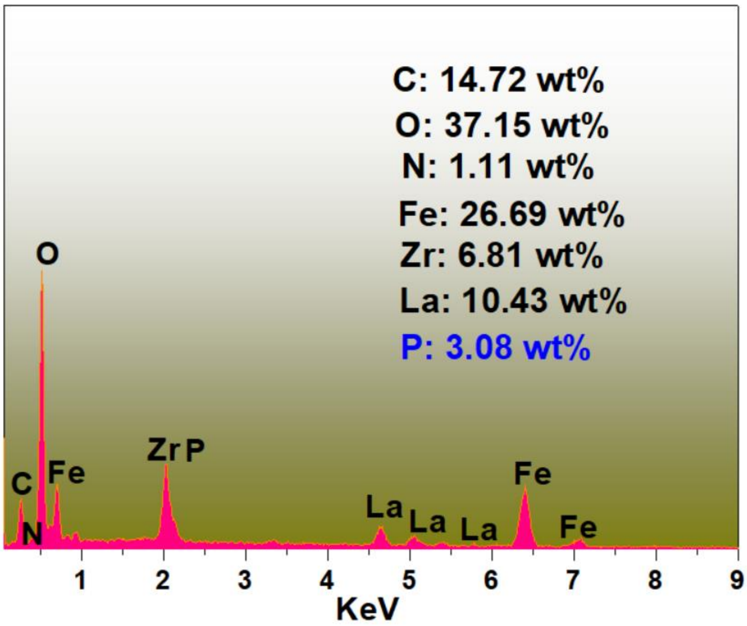

Figure S7. EDX spectrum of phosphate adsorbed-material.

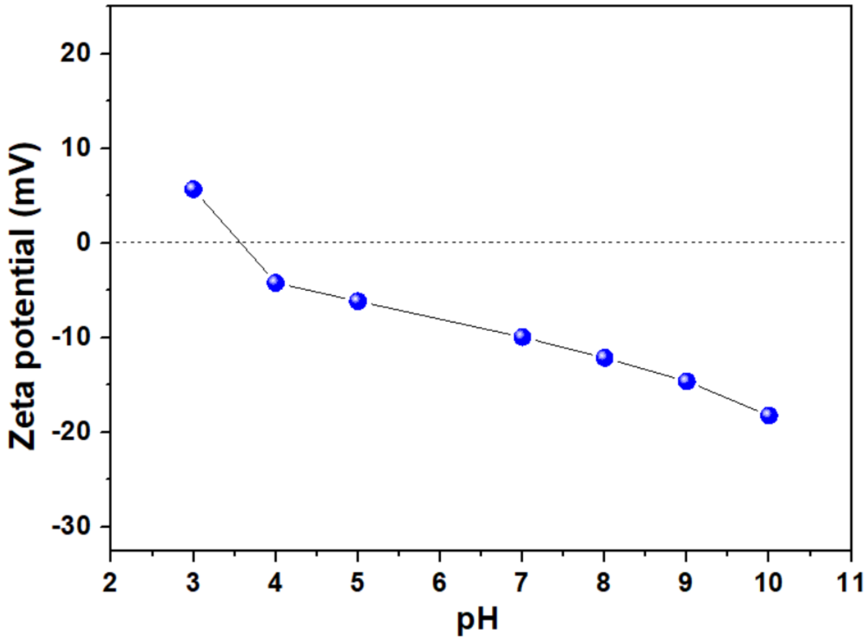

Figure S8. Zeta potential in response to pH change.
